# Editorial: Outstanding advances in veterinary diagnostic ultrasonography: novel milestones in disease detection, prediction, and treatment, volume II

**DOI:** 10.3389/fvets.2026.1977271

**Published:** 2026-09-16

**Authors:** Hussein M. El-Husseiny, Khan Sharun, Ryou Tanaka

**Affiliations:** 1 Institute of Global Innovation Research, Tokyo University of Agriculture and Technology, Tokyo, Japan; 2 Veterinary Teaching Hospital, Tokyo University of Agriculture and Technology, Tokyo, Japan; 3 Department of Surgery, Anesthesiology, and Radiology, Faculty of Veterinary Medicine, Benha University, Toukh, Egypt; 4 Regenerative Medicine Group, Department of Health Science and Technology, Aalborg University, Gistrup, Denmark

**Keywords:** AI, artificial intelligence, diagnostic imaging, echocardiography, ultrasonography, veterinary practice

Currently, ultrasonography (US) is widely used as an efficient diagnostic tool in veterinary practice. It has been proven to be superior over radiography in clearly visualizing internal organs and detecting diverse conditions. This enabled the precise assessment of the anatomy and function of various body organs, whether they are healthy or diseased. Furthermore, US could be used efficiently to provide qualitative evaluation and quantitative scoring of the healing process following surgeries. In addition, color Doppler US has been used to assess the function of various organs by evaluating their vascular hemodynamics. The adoption of novel technologies in US marked a milestone that extends its applications to the early detection and even prediction of many diseases, especially cardiac conditions. This advancement consequently aids in designing and assessing prompt therapeutic protocols and minimizing possible deaths.

The present Research Topic aimed to highlight the existing progress in diagnostic US and emphasizing its diverse and promising applications in veterinary practice. It includes the most substantial and recent research conducted on various large and small animal models, as well as the use of artificial intelligence in advancement of diagnostic imaging. The present Research Topic comprised six published articles that align with these strategies.

Park et al. evaluated the utility of US in the assessment of adrenal glands in 38 clinically normal Korean raccoon dogs (*Nyctereutes procyonoides koreensis*). They aimed to establish species-specific reference values for the adrenal gland measures in these dogs, which are important for veterinary practice and wildlife medicine. A retrospective analysis of sagittal US included adrenal length, height of the cranial pole, and height of the caudal pole of both left and right glands. Moreover, the morphology and corticomedullary distinction were visually evaluated. Ultrasonography enabled the clear visualization of the adrenal glands without sedation. In conclusion, this study presented the first ultrasonographic reference values for the adrenal glands in clinically healthy Korean raccoon dogs. The morphology and adrenal size were consistent across sex and body weight, supporting the use of a single reference range for adults. These findings will improve the diagnostic precision of US in veterinary imaging and support species-specific evaluation in wildlife medicine.

Martinez et al. studied the feasibility of deep learning AI models to assist in the diagnosis of life-threatening conditions—specifically effusions (fluid accumulation) and pneumothorax (PTX, air in the chest cavity)—in dogs using point-of-care ultrasound (POCUS). They aimed to improve the diagnostic accuracy, particularly in settings where expert operators are unavailable, such as military or remote environments. A total of 25 dogs diagnosed with PTX were enrolled in this investigation. Ultrasound clips were collected from diverse anatomical areas using standardized protocols. Then, the images were reviewed, curated, and labeled by experts. To develop the AI model, multiple convolutional neural network (CNN) architectures were trained using transfer learning from large pre-existing medical imaging datasets. The models were optimized and evaluated using a leave-one-subject-out cross-validation approach to confirm generalizability. Moreover, to address dataset limitations, image augmentation and class balancing techniques were applied. In conclusion, AI-assisted ultrasound interpretation is feasible and promising for augmenting veterinary emergency triage, particularly in military and remote applications. With further development, these AI models could help first responders and less experienced practitioners detect life-threatening conditions in dogs, ultimately improving early intervention and outcomes.

Transducer (probe) selection is often operator-dependent, with limited evidence-based guidelines in veterinary practice. Granger et al. provided an evidence-based review article on the optimized selection of US transducers for the diagnosis of alveolar-interstitial syndrome (AIS), a common lung ultrasound (LUS) finding in small animals with various pulmonary diseases [e.g., pulmonary edema, pneumonia, contusions, acute respiratory distress syndrome (ARDS)]. They reviewed the present literature in human and veterinary medicine to investigate how probe choice can affect the diagnosis and imaging of AIS in dogs and cats. They found that all major transducer types can detect AIS in small animals; however, the selection should be adjusted to the patient's size, clinical objectives, and the equipment available. Microconvex probes are generally preferred for versatility; meanwhile, linear probes are best for detailed pleural imaging. Moreover, it was concluded that consistency in transducer use is crucial for monitoring disease progression. Standardization and further studies will enhance diagnostic accuracy and patient outcomes in veterinary emergency and critical care.

Injuries of the intermediate patellar ligament (IPL) in horses present a high clinical significance, and traditional US often yields non-specific findings. Hence, Straticò et al. investigated the use of two advanced US techniques: strain elastography (SE) and two-dimensional shear wave elastography (2D-SWE) to assess the IPL in horses. Longitudinal and transverse SE scans to measure the strain ratio (SR) between the IPL and the infrapatellar fat pad and the elasticity index of the IPL (EI_IPL_), in addition to 2D-SWE scans to measure the Young's modulus (kPa) and shear wave velocity (m/s), were used to assess the IPLs in sound and lame horses. Evaluating the same region of interest (ROI) revealed that both techniques were feasible. They concluded that SE and 2D-SWE could efficiently study equine IPL and could potentially be used together with the conventionally used techniques such as X-ray and US. Moreover, these tools could differentiate the elastic characteristics of equine IPLs under normal and chronic injury conditions. However, marked limitations of this study are still to be addressed in future investigations, including the employment of Doppler US to further investigate the vascular structure of the ligaments in a larger number of studied animals.

Conventional echocardiography was utilized to provide quantitative evaluation of the severity of mitral regurgitation (MR) in dogs with myxomatous mitral valve disease (MMVD). Kawai et al. conducted a retrospective study of 167 MMVD small-breed dogs categorized based on the staging system of the American College of Veterinary Internal Medicine (ACVIM). They found that quantitative regurgitation indices such as regurgitant volume normalized to body weight (RVol/kg) and regurgitant fraction (RF) were significantly correlated with conventional echocardiography measures in MMVD dogs. Moreover, they could provide valuable insights on the severity of MR, structural evaluation, and staging of the disease. Furthermore, the mitral-aortic velocity-time integral (VTI) ratio was strongly associated with regurgitation measures and provided an outstanding ability to diagnose and quantify the severity of MR. The authors concluded that conventional echocardiography, combined with the calculation of quantitative regurgitation indices, is a promising tool for evaluating MR, although further investigations are still required to validate these findings.

In an exciting case report, Tsuka et al. used US in combination with computed tomography (CT) to study one of the rare vascular anomalies named Juvenile angiomatosis in bovine practice for the first time. The 4-day-old male Japanese-Black calf was presented with different swollen lesions on the dorsal and ventral aspects of the thoracic skin on the left side. US examination showed the presence of structures with homogeneous echogenicity in the subcutaneous space, with poor vasculature confirmed with Doppler US. The findings were confirmed, counted, and characterized using CT. Moreover, the structure of the masses was assessed by histopathology and immunohistochemistry for von Willebrand factor and alpha-smooth muscle actin. However, US and CT provided a preliminary diagnosis lacking detailed characterization of the masses. The combined diagnostic scheme, including US, CT, histopathology, and immunostaining, could provide a detailed examination and a definitive diagnosis for such rare cases.

Taken together, the articles in the present Research Topic provide a coherent and proactive perspective on US as a key element of veterinary diagnostic imaging. They show that US is a potent diagnostic tool for diverse conditions affecting different organs and systems across multiple animal species. The field is moving toward a more thorough and integrative diagnostic model by linking imaging results to clinical outcomes, utilizing cutting-edge technology, and modifying methods for underrepresented species. Moreover, the integration of AI in future research will pave the way for further outstanding advancements.

